# Book Reviews

**Published:** 1874-12-01

**Authors:** 


### Book Reviews.

CYCLOPEDIA OF THE PRACTICE OF MEDICINE. Edited by H. Von Ziemssen, Prof. of Clinical Medicine, Munich, Bavaria. Vol. 1, Acute Infectious Diseases, by Prof. Liebermeister, of Tubingen, Prof. Liebert, of Breslau, Dr. Haenisch, of Greifswald, Prof. Heubner, of Leipzig, and Dr. Oertel, of Munich. Albert H. Buck, Editor of American Edition. New York: Wm. Wood & Co. Sold only by Subscription. W. T. Keener, Agent, 94 Washington Street, Chicago.

Will be reviewed in an early number of the EXAMINER.

---

TRANSACTIONS OF THE INDIANA STATE MEDICAL SOCIETY, 1874.

A handsome volume of 220 pages, cloth covers, containing numerous valuable and interesting essays.

---

TRANSACTIONS OF THE MEDICAL SOCIETY OF THE STATE OF PENNSYLVANIA. Twenty-fifth Annual Session, held at Easton, Pa., May, 1874. Pp. 450.

---

TRANSACTIONS OF THE NEW HAMPSHIRE STATE MEDICAL SOCIETY. Eighty-fourth Anniversary, held at Concord, June 9th, 1874.

---

THE LEGAL RELATIONS OF EMOTIONAL INSANITY. By L. Howard, M.D., of Baltimore, Md. Extracted from the transactions of the American Medical Association.

---

TREATMENT OF ZONA BY COLLODION AND MORPHIA.—Dr. Bourdon, Hopital la Charite, after having tried a great many local means for treating the above disease, and checking the intense pain, has definitively adopted the following plan:—Without opening the vesicles, he paints all the diseased surface with a combination of collodion and morphia—collodion one ounce, morphia eight grains. The mixture must be put on pretty thickly. The pain ceases from the second day, and at the end of seven or eight days, when the layer of collodion is removed, all the vesicles have disappeared, and there remains only a slight local redness.—*Canada Lancet.*

---

PETRIFACTION VERSUS CREMATION.—Dr. Steinbeis, of Wurtemburg, proposes to dispose of the dead by placing the body in a trough of cement, and then filling the space with liquid cement, which will harden and convert the whole into a solid mass of stone. The blocks thus obtained may be piled up, buried, or inscribed and set up to do duty as both tombs and tomb-stones. This method, if generally adopted, possesses some advantages for posterity, as future generations would probably use the obsolete blocks for building material.—*New York Medical Journal.*

---

DR. HAMBERG, of Stockholm, has lately been engaged in making a series of experiments on the poisons exhaled by certain wall papers. As with ourselves, arsenic is the poison most to be dreaded.—*London Lancet.*

---

OFFICE AND PRACTICE FOR SALE.—A good Office and a comfortably furnished Cottage and a complete stock of Drugs, are offered for sale very cheap, with the succession to a practice guaranteed to be worth three thousand dollars per annum. Good reasons for selling.
Address J. E. LEWIS, M.D.,
Fulton, Ark.

LOCATION WANTED.—A Physician, who has been in practice about three years, and is now Assistant Physicion in a State Hospital for the Insane, desires to find a favorable location for private practice. Communications may be addressed to W. B., care of publishers MEDICAL EXAMINER, 65 East Randolph St., Chicago.

